# Osmotolerance is a driver of microbial carbon processes in the Elbe estuary

**DOI:** 10.1128/msystems.01790-25

**Published:** 2026-03-30

**Authors:** Sven P. Tobias-Hünefeldt, Jason N. Woodhouse, Hans-Joachim Ruscheweyh, Shinichi Sunagawa, Vanessa Russnak, Wolfgang R. Streit, Hans-Peter Grossart

**Affiliations:** 1 Department of Plankton and Microbial Ecology, Leibniz Institute of Freshwater Ecology and Inland Fisheries (IGB), Stechlin, Germany; 2 Department of Microbiology and Biotechnology, Institute for Plant Sciences and Microbiology University of Hamburg 14915, Hamburg, Germany; 3 Molecular Systems Biology Unit, European Molecular Biology Laboratory 9471, Heidelberg, Germany; 4 Department of Molecular Animal Physiology, Institute for Animal Cell and Systems Biology, Hamburg, Germany; 5 Department of Biology, Institute of Microbiology and Swiss Institute of Bioinformatics, ETH Zürich, Zürich, Switzerland; 6 Institute of Carbon Cycles, Helmholtz Centre Hereon 28338 , Geesthacht, Germany; 7 Institute of Biochemistry and Biology, Potsdam University, Potsdam, Germany; University of Illinois Chicago, Chicago, Illinois, USA

**Keywords:** estuary, salinity gradient, carbon cycle, metagenomes, metatranscriptomes, particle-associated vs. free-living bacteria

## Abstract

Estuaries are blue carbon loci, storing and exchanging carbon between aquatic, atmospheric, and terrestrial environments. Estuarine particles facilitate the transformation and transport of organic matter. The fate of particulate organic matter in estuaries is driven by structural changes in polymers that modify buoyancy, determining the proportions of sinking and suspended particles. In the open ocean and coastal ecosystems, the microbial composition and function of sinking and suspended particles differ, impacting carbon remineralization and sedimentation rates. We leverage 190 metagenomes and 73 metatranscriptomes to assess free-living, sinking, and suspended particle-associated microbial composition and function across the Elbe estuary. The salinity gradient in the Elbe estuary is the primary driver of microbiome composition and function. Transparent exopolymer particles (TEP) production was localized to freshwater, with seemingly no TEP-associated organisms detected above 20 practical salinity units (PSU). We observed differences in the function of free-living and particle-associated microbial communities, with diazotrophs enriched on particles. We observed that sinking particles may better support methanogenesis, and suspended particles showed signs of continued primary and secondary production. From this, we conclude that activities such as dredging, which resuspend sediment, will exacerbate carbon turnover and greenhouse gas emissions, and reduced dredging may lower greenhouse gas (GHG) emissions in the Elbe estuary. Many of these GHG linking processes are inhibited by salinity due to the osmosensitivity of methanogens and methanotrophs along the estuary. Changes in sea level and precipitation rates will likely directly interact with activities such as dredging, with as yet uncertain impacts on microbial carbon processing and storage.

**IMPORTANCE:**

Estuaries, lower river areas that merge into oceans, play a large role in Earth’s carbon cycle. Estuaries store carbon and manage greenhouse gases, exchanging carbon between land, water, and the air. As carbon travels down estuaries, it is processed by free-living and particle-associated microbes. We explore the relationship between environmental conditions and present and expressed genes. Based on gene profiles, methane concentrations in the water column may be related to the abundance of sinking particles, while suspended particles are linked to growth and energy acquisition. Therefore, the balance of suspended vs. sinking particles is important in highly turbid estuaries, like the Elbe estuary, where urban activities affect greenhouse gas emissions and salinity intrusions. Dredging often tips the balance toward sinking particles and therefore increased greenhouse gas emissions. Our study thereby informs future policy decisions and the impact these decisions will have on our future climate.

## INTRODUCTION

Aquatic environments act as carbon dioxide (CO_2_) storage and processing centers, as well as organic matter (OM) production, utilization, and transportation hubs (1). These ecosystems link terrestrial and oceanic ecosystems (2) and drive terrestrial-atmosphere transfers (3). In particular, estuaries represent critical environmental carbon hotspots, remineralizing >70% of terrestrial organic matter entering riverine ecosystems. This remineralization is primarily driven by microorganisms, particularly those colonizing particles (4, 5). Thus, it is critical to understand how microbial processes might be impacted by changes in the structure of organic matter, salinity, and temperature. Estuaries are characterized by distinct salinity gradients and dynamic environmental conditions, including freshwater flow and marine intrusions, which shape their biodiversity, heterotrophy rates, food-web efficiency, and nutrient/carbon transport (6, 7). These gradients therefore represent dynamic conditions based on tidal, freshwater, and marine influences (8) and may even create a barrier that freshwater organisms cannot overcome. As a consequence, phytoplankton bloom intensity and productivity may be spatially constrained. Spatial restrictions leading to localized oxygen depletions following bloom collapse, with hypoxic area prevalence and size increasing over time (9, 10). The oxygen depletion creates a hostile environment, killing or isolating endemic species and shifting ecosystem conditions, e.g., redox conditions affecting carbon and nitrogen cycling. For instance, during productive seasons (i.e., spring and summer), estuaries can act as carbon sinks. Yet, high CO_2_ freshwater production rates lead to a net annual CO_2_ production (11). This shows how estuaries are highly salinity-dependent (12, 13) and play a key role in greenhouse gas emissions and climate change feedback (14). Moreover, biogeochemical dynamics and carbon exchanges are altered by intense human activities and climate change, leading to pollution and eutrophication, further intensifying hostile conditions, e.g., anoxia and the presence of hydrogen sulfide. Consequently, estuaries represent dynamic and complex ecosystems where physical, chemical, and biological processes are highly interdependent, shaping biota and their related biochemical processes.

The Elbe estuary (Germany) has a catchment of 140,268 km², making it the second largest German River to discharge into the North Sea (15), and represents an important system for the analysis of organic matter dynamics (16). The Elbe estuary supports a wide range of species and habitats but suffers heavy anthropogenic impacts, e.g., shipping and industrial activities. Numerous attempts have been made to understand the source and fate of inorganic/organic carbon in the Elbe estuary (16–18), with an outsized focus on freshwater zones (11, 19, 20).

Particles represent major carbon sources in estuarine ecosystems and are mobilized proportional to discharge conditions. Particles transport carbon between pelagic, benthic, and aquatic environments with sequestration in estuarine and marsh sediments. Differences between suspended and sinking particles have been identified in the Wadden Sea, and within the Elbe estuary itself (16, 21). Elbe estuary particles differ in density and particulate organic carbon (POC) abundance and are linked to either marine-like (sinking) or terrestrial-like (suspended) humic-like dissolved organic matter (DOM). However, Elbe particles are highly variable due to the many influences acting on aggregation, i.e., anthropogenic influences such as dredging to maintain channel depth for ship traffic, marsh exchanges, and seasonal phytoplankton and zooplankton abundances. Therefore, particles differ significantly between upper and lower Elbe estuary regions, with dredging focusing on lower regions, resulting in resuspended sediments increasing turbidity for 100s1,000s of meters and driving community changes (22, 23).

Particle degradation and DOM turnover are closely related to bacterial OM transformations, with microbial particle degradation representing a major source of CO_2_ (24, 25) and an essential biogeochemical driver (26, 27). Individual estuarine effects (i.e., the associated nutrient/salinity gradient) on microbial particle degradation are well-assessed (28), with shifts in the estuarine gradient associated with particle-associated community composition and colonization responses (16, 20). However, the microbiome composition represents only one aspect of the numerous variable factors. It is increasingly necessary to integrate physicochemical profiles, metaOMICs approaches, DOM profiles, and particle characteristics to gain a more comprehensive overview of turbid estuaries and how microorganisms are involved in carbon processing (29–31).

To better understand the role of microorganisms in carbon processing in the Elbe estuary, we performed regular sampling along five sites in the Elbe estuary between Hamburg and the North Sea. These data allow us to explore spatiotemporal patterns across the turbid Elbe estuary. Specifically, comparing microbial community compositions and functions between particle-associated and free-living microbiomes while integrating particulate (POC) and dissolved organic carbon (DOC) dynamics. We hypothesized that freshwater areas will contain organisms that lack osmoregulation genes, spatially constraining taxa that perform key roles in biogeochemical cycling of organic and inorganic carbon. Salinity-dependent localization is expected to represent the primary driver of microbiome composition and function in the estuary and explain the absence of processes (e.g., nitrification) in the brackish and marine sites (32). Our data also enable a detailed analysis of free-living vs. particle-associated lifestyles in a highly turbid and turbulent estuary (33). We hypothesized that particle-associated microbiomes play a greater role in complex polymer degradation than free-living bacteria. Specifically, we expected that suspended particles are enriched for organisms involved in labile organic matter degradation such as amino acid biosynthesis, whereas sinking particles are preferentially linked to recalcitrant aromatic compound degradation.

## MATERIALS AND METHODS

### Sampling

**Fig 1:**
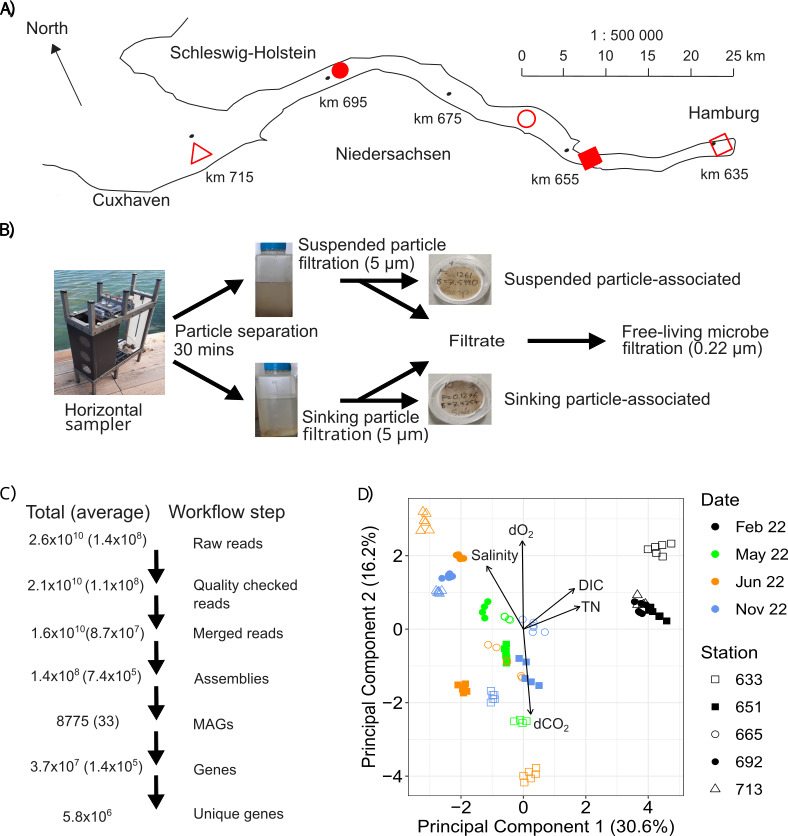
Elbe estuary map. The map (**A**) depicts the outline of the Elbe estuary, from Hamburg Harbor to Cuxhaven, with marks of the Elbe estuary length (in kilometers) shown as black circles. Sample stations are depicted as red shapes, used throughout the manuscript. The sampling workflow for microbial communities is depicted (**B**) with pictures depicting the horizontal sampler, suspended/sinking particle separation, and filtration results. The sequencing workflow (**C**) with the total and average number of raw, quality-checked, and merged reads, assemblies, MAGs, and genes per sample. The PCA (**D**) depicts environmental changes across the study period during sample acquisition; only 2,022 samples are shown due to their complete physicochemical profiles, excluding 2,021 samples. The top five contributors to sample dissimilarity are shown. Adapted from Tobias-Hünefeldt et al. (16).

Samples were taken from the River Elbe estuary in the main channel, Germany, in May 2021, July 2021, February 2022, May 2022, June 2022, and November 2022 at 5 stations (Mühlenberger Loch [53.54907, 9.82338], Twielenfleth [53.60921, 9.56536], Schwarztonnensand [53.71442, 9.46976], Brunsbüttel [53.88742, 9.19429], and Medemgrund [53.8363, 8.88777]; Fig. 1A). Samples were taken with a horizontal sampler (21) at a depth of 1 m. Due to very high turbidity in the Elbe estuary caused by continuous dredging, primary production rapidly decreased. Suspended and sinking particles were allowed to vertically separate for 30 minutes (Fig. 1B) and filtered separately to facilitate independent analyses. A 30-minute settling time to separate suspended and sinking particles was chosen based on Lunau et al. (21), and a mean current velocity of <5–76 cm s^−1^, dependent on ebb-flow cycle and season (21, 34). Particle-associated microbiomes were captured on 5 µm Durapore filters, the filtrate used to capture free-living microbes on 0.22 µm Durapore filters, for a total of three fractions: suspended particle-associated, sinking-particle-associated, and free-living. A total of six time points were assessed, with all samples collected in duplicate, unless otherwise stated. Each time point was represented with 30 discrete water samples from the Elbe estuary, representing five sites (Fig. 1A), with six samples per station (two free-living, two suspended particle-, and two sinking particle-associated; Fig. 1).

Particle characteristics were assessed by filtering samples onto pre-weighed and rinsed GF75 ADVANTEC filters, followed by another rinsing to remove salts. Characteristics included particulate matter dry weight and particulate total and organic carbon contents (PTC and POC). Before use, filters were pre-combusted for 5 hours at 400°C to remove any residual carbon. A total of 20 filters were measured per time point, including suspended and sinking particle fraction duplicates.

### DNA extraction and sequencing

DNA was extracted using the method described in Nercessian et al. (35). In brief, cells were lysed with zirconia-silica beads (0.1–1 mm) suspended in cetyltrimethyl ammonium bromide (CTAB). Sodium dodecyl sulfate and N-lauroylsarcosine (anion surfactants), proteinase K, and phenol-chloroform-isoamyl alcohol were added. Chloroform-isoamyl alcohol and polyethylene glycol (PEG) were used for DNA purification. DNA was precipitated at 4°C, washed with ethanol, air-dried, and finally dissolved in Tris (Tris-hydroxymethyl-aminomethane). Metagenomic sequencing was performed at the Ramaciotti Centre for Genomics (Sydney, Australia) and the Competence Centre for Genomic Analysis Kiel (Kiel, Germany). Samples were prepared for sequencing with the Illumina DNA Prep Kit and sequenced on a NovaSeq 6000 platform (Illumina, San Diego, CA, USA).

Although 210 metagenome and 90 metatranscriptome samples were collected, not all samples contained sufficiently high-quality sequences (>10M × 150 bp reads with a Phred score >30). Therefore, only 190 metagenomic samples and 73 metatranscriptomic samples, including duplicates, were processed. Sequences were processed as outlined in reference (36) with a detailed description available in the Supplemental Methods. In brief, sequences were quality filtered with BBMap (v.38.79; https://sourceforge.net/projects/bbmap/), metagenomes were assembled with metaSPAdes (v.3.15.2 [37]), reads mapped to scaffolds with BWA (v.0.7.17-r1188 [38]), and MetaBAT2 (v.2.12.1 [39]) was used to quantify depth and bin individual metagenome samples. Metagenome bin qualities were assessed with CheckM (v.1.1.3 [40]) and Anvi’o (v.7.1 [41]), keeping bins above ≥50% completeness/completion (cpl) and a contamination/redundancy (ctn) of ≤10%, in accordance with best practice. MAG taxonomy was assigned with GTDB-Tk (v.2.1.0 [42]) against the GTDB R214 release (43) and gene sequences predicted with Prodigal (v.2.6.3 [44]).

Genes were clustered at 95% with CD-HIT (v4.8.1 [45]) and aligned and classified against Kyoto Encyclopedia of Genes and Genomes (KEGG; v.2019-03-20; https://www.genome.jp/kegg/ [46–48]), Pfam (v.32 [49, 50]), and VOG (release 94 [51]) databases using DIAMOND (v2.0.15 [52]). Gene-length normalized read abundances were converted to per-cell gene copy number using single-copy marker genes (53). Meanwhile, rRNA sequences were extracted with Barrnap (v.0.9; https://github.com/tseemann/barrnap), and taxonomic profiling extended the mOTUs database (v.3.1 [53]) with 13,765 prokaryotic MAGs. A total of 1,046 mOTUs (marker gene-based operational taxonomic units) were identified from Elbe metagenomes, 1,202 mOTUs from the extended database, and 778 mOTUs matching previous reference sequences. Additional MAG dereplication was performed with dREP (v.3.0.0 [54]).

Total and median read and gene abundances, as well as MAG numbers, are displayed in Fig. 1C. With an emphasis on genes involved in monomeric and polymeric organic carbon substrate processing, we established an extensive genome and gene catalog to understand shifts in composition and function of free-living, suspended, and sinking particle-associated microbiomes. The CO_2_ and methane (CH_4_) gene database was manually curated based on the KEGG database (46–48), extracting all CO_2_ (C00011) and CH_4_ (C01438) associated genes. Potential osmoregulation genes were classified based on KEGG annotations or prior identification in Jurdzinski et al. (55) if a potential osmoregulation mechanism was established.

### Physicochemical parameters

Salinity, oxygen, temperature, and turbidity were measured with the onboard FerryBox (56), and bulk water utilized to measure dissolved CO_2_ and CH_4_ concentrations using the headspace technique. In brief, 100 mL of water was collected in a 500 mL syringe, and 400 mL of pure N_2_ gas was added, vigorously shaking the syringe for 1 minute. Transferring 350 mL of gas to 1 L gas bags, measuring CO_2_ and CH_4_ concentrations within 12 hours using an Ultraportable Los Gatos (Los Gatos Research, USA), as described in Kang et al. (57). POC, PTC, DOC, dissolved ammonium, dissolved nitrate, dissolved nitrite, soluble reactive phosphate (SRP), and total dissolved nitrogen, phosphate, and silicate were determined as outlined in Tobias-Hünefeldt et al. (16).

### Statistical analysis

All codes associated with statistical tests, intermediate files, manually modified files, and figure creation are available at https://github.com/SvenTobias-Hunefeldt/ElbeMicrobiome. All figures were generated with ggplot2 (v.3.5.1 [58]) and finalized with Inkscape (v.1.3.2), unless otherwise stated. The prcomp() function from the stats package (v.4.1.2) was used to generate the physicochemical PCA figure. Group differences were assessed with the ANOSIM and PERMANOVA tests with the vegan package (v.2.6-6.1), and ANOVA tests from the stats package; eta_squared() from the rstatix package (v.0.7.0) assessed the correlation strength of ANOVA tests. Differences between two group means were assessed with wilcoxon.test() from the stats package, and pairwise Wilcoxon tests from the stats vegan package identified pairwise differences. Spearman tests with cor.test() from the stats package assessed correlations between two trends. Network analyses were carried out with the WGCNA package (v.1.72-5) in R, and excluded genes from the network are removed from subsequent statistical analyses. Individual modules were determined by generating a co-occurrence Pearson correlation network, where each taxa/gene’s weighted correlation coefficient determined groups according to hierarchical clustering, where each branch represents individual modules. WGCNA (weighted gene co-expression network analysis) network construction used a deep split of 2, a merge cut height of 0.25, and a maximum module size of 40,000. The functional potential WGCNA network had a soft-threshold power of 10 during construction, and a minimum module size of 50, like the transcript per gene network. Meanwhile, the mOTU network construction used a minimum module threshold of 15. Transcript per gene and mOTU networks used soft-threshold powers of 5 and 7, respectively. Network eigenvalues per cluster were used to determine module correlations with physicochemical measurements, and mOTUs were linked to functional traits via their corresponding MAGs, if available. Mantel tests from the vegan package (v.2.6-6.1) compared distance matrices [generated using dist()] between mOTUs, metagenomes, and transcripts per genome, and their network module eigenvalues. Metatranscriptome-derived mOTUs could not be utilized, due to the low number of matches between metatranscriptomes and marker genes; therefore, all mOTUs are derived from metagenomic data.

## RESULTS AND DISCUSSION

**Fig 2:**
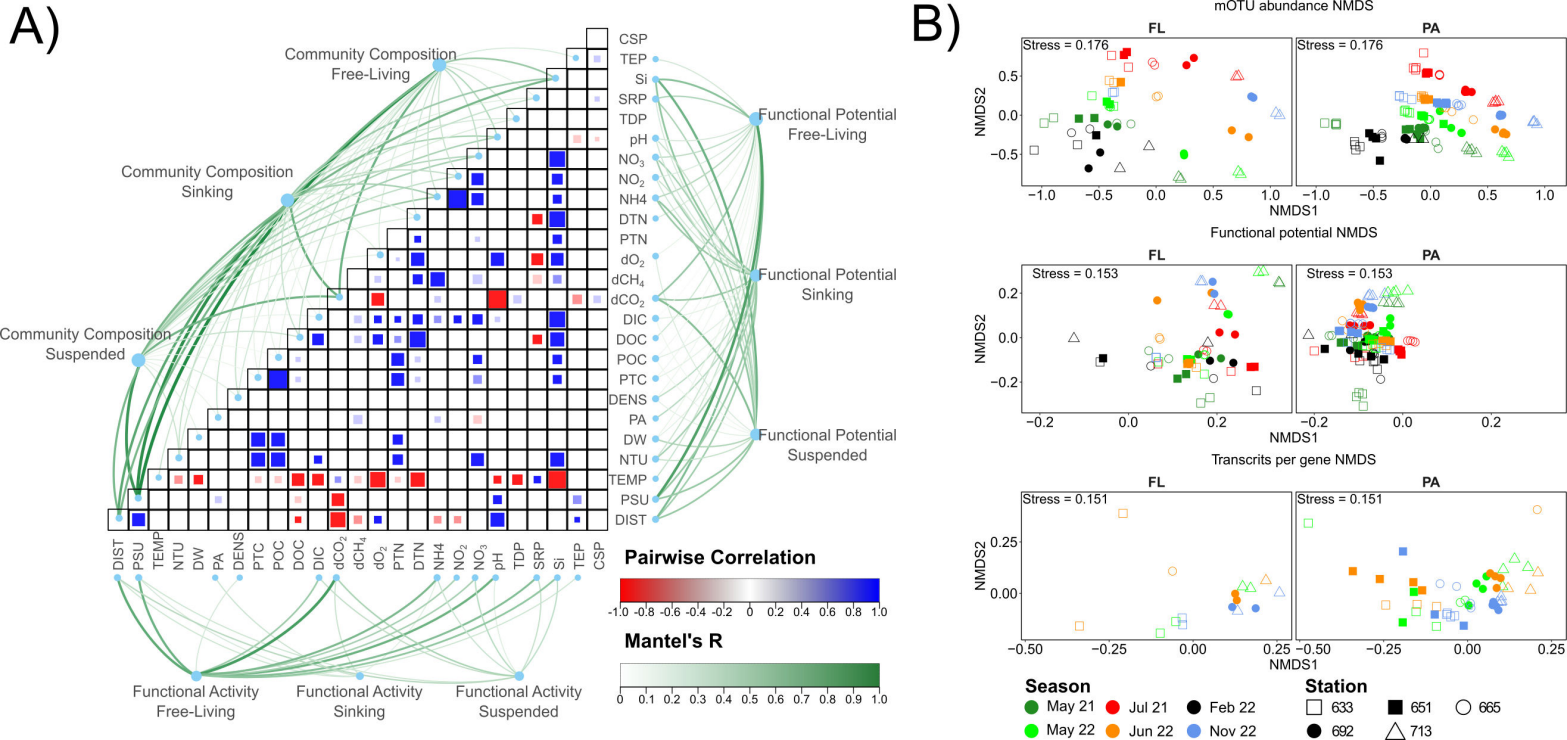
Microbiome aspects in the ecosystem. Pairwise correlations between the microbial community composition, functional potential, and transcription profiles (**A**) using Mantel tests. Colors represent the pairwise correlation, and alpha the Mantel’s *R* value. Non-significant interactions (corrected *P* > 0.05) are not shown. DIST= Elbe estuary kms, PSU = salinity (practical salinity units), TEMP = temperature (°C), NTU = turbidity (nephelometric turbidity units), DW = particulate dry weight (mg L^−1^), PA = particle area (µm^2^ L^−1^), DENS = particle density, PTC = particulate total carbon (mg L^−1^), POC = particulate organic carbon (mg L^−1^), DOC = dissolved organic carbon (mg L^−1^), DIC = dissolved inorganic carbon (mg L^−1^), dCO_2_ = dissolved CO_2_ concentration (µM), dCH_4_ = dissolved CH_4_ concentration (µM), dO_2_ = dissolved oxygen concentration (µM), PTN = particulate total nitrogen (mg L^−1^), DTN = dissolved total nitrogen (mg L^−1^), NH_4_ = dissolved ammonium (mg L^−1^). NO_3_ = dissolved nitrate (mg L^−1^), NO_2_ = dissolved nitrite (mg L^−1^), TDP = total dissolved phosphate (mg L^−1^), SRP = soluble reactive phosphate (mg L^−1^), Si = dissolved silicate (mg L^−1^), TEP = transparent exopolymer particles (µm^2^ L^−1^), CSP = Coomassie stainable protein (µm^2^ L^−1^). (**B**) NMDs plots of microbiome mOTUs, functional potential, and transcripts per gene, with free-living (FL) and particle-associated (PA) samples separated. Colors represent sample dates and shape sample stations along the Elbe estuary. Outliers have been removed.

Measured environmental variables (i.e., temperature, salinity, nutrients, DOC quantity and quality, POC quantity and quality, CO_2_, etc.) exhibited a high degree of spatiotemporal variation (Fig. 1D) (16). Many of these parameters (i.e., salinity, dissolved CO_2_ [dCO_2_], and pH) exhibited strong spatial patterns and correlated with their spatial location along the Elbe estuary (km), whereas seasonality was primarily associated with temperature, dissolved nutrients, and turbidity (Fig. 2A). Spatiotemporal patterns showed that seasonality was a stronger driver of carbon dynamics than salinity, strong mixing forces, and high particle heterogeneity obscuring the expected spatial patterns. Particles were smaller and more abundant than previously seen (11), ranging in size and abundance from 2 × 10^5^ µm^2^ L^−1^ to 7 × 10^7^ µm^2^ L^−1^. While less relevant to carbon dynamics, salinity, turbidity, and dissolved oxygen, carbon dioxide (CO_2_), and methane (CH_4_) retained spatial differences between upper and lower estuary regions (Fig. S1) (16).

Mantel tests were performed to correlate environmental data to variation in community composition, functional potential, and gene expression. Significant differences between free-living and particle-associated (made up of both suspended and sinking particle-associated fractions) microbiomes were evident at the level of community composition and functional potential (Fig. 2B; Table S1; PERMANOVA, *P* < 0.01). Free-living vs. particle-associated differences are long established and predominantly based on particle-associated microorganism’s increased metabolic potential and activity compared to free-living microorganisms (59–61).

Contrary to our predictions, no significant differences are present between sinking and suspended particle microbiomes (Fig. 2B, *P* > 0.05). We hypothesized that differences in the specific properties of the two particle types (carbon and nitrogen content, buoyancy, and proportions of TEP and Coomassie stainable particles [CSP]) would result in differences in the microbiome community composition and function, based on biochemical differences observed in the Wadden Sea (62) and Elbe estuary (16). Instead, the Elbe’s high turbulent kinetic energy likely minimizes differences. Strong tidal forces and high flow rates keep particles in suspension with irregular aggregation-disaggregation dynamics. This effect is especially pronounced in the Elbe estuary’s turbidity maximum zone (TMZ), where turbulent kinetic energy is up to 240 times higher than in the Wadden Sea (33, 62), where significant particle differences were previously seen (21). Our findings agree with others suggesting that differences are shaped less by microbial composition or functional potential than colonization rates and particle density (63, 64).

### Salinity and osmoregulation genes drive dissolved CO_2_ and CH_4_ gene profiles

Spatiotemporal differences consistently influence environmental conditions (Fig. 1C), with strong temporal differentiation between all samples (ANOSIM, *R* > 0.37, *P* < 0.01; PERMANOVA, *R*^2^ > 0.31, *P* < 0.01). In contrast, transcription profiles exhibit weaker temporal patterns between all samples (Fig. 2; ANOSIM, *R* = 0.13, *P* < 0.01; PERMANOVA, *R*^2^ = 0.09, *P* < 0.01), while spatial partitioning is more pronounced (ANOSIM, *R* = 0.34, *P* < 0.01; PERMANOVA, *R*^2^ = 0.41, *P* < 0.01). Although reduced temporal patterns may reflect both the aggregation of KEGG Orthology terms with functional redundancy over time (65) and a strong spatial selection pressure of key gene expressions along the estuary gradient (66).

Salinity is the dominant microbiome driver, strongly correlating with high community composition and functional potential (Fig. 2; Mantel, *R* = 0.37–0.75, *P* < 0.01), though less so with transcription profiles (Mantel, *R* = 0.19–0.51, *P* < 0.01). Its variability, driven by marine intrusions, tides, and upstream flow rates, complicates clear spatial and temporal separation. Consequently, composition and functionality differ significantly between mesohaline/polyhaline (downstream) and oligohaline/freshwater (upstream) sites, with fluctuating salinity content at Schwarztonnensand. Spatial analyses were therefore based on salinity levels, rather than stations.

**Fig 3:**
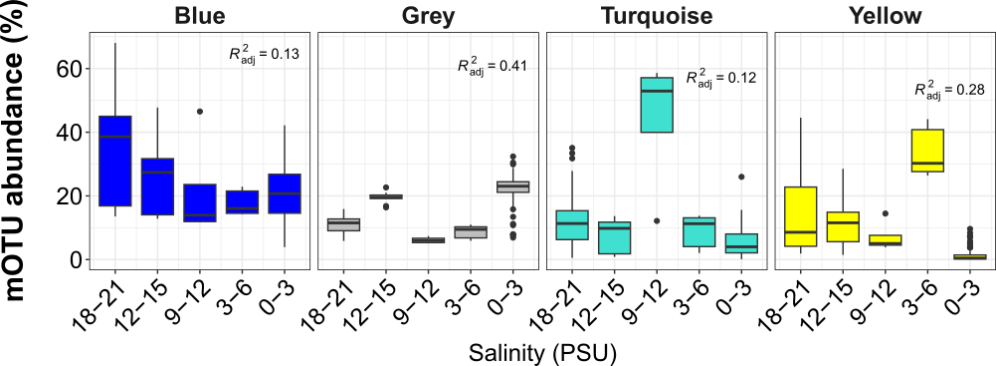
WGCNA mOTU module abundance in relation to salinity. Total module mOTU abundance is shown in against salinity, binned every 3 PSU, with an adjusted *R*^2^ value.

Weighted gene co-expression network analysis (WGCNA) identified four salinity-correlated mOTU (marker gene-based operational taxonomic units) modules (Fig. 3; Fig. S2 and S3). The gray module, which declines with salinity, is predominantly composed of freshwater and non-salinity-associated taxa such as *Limnohabitans* (67), *Pseudoholngiellaceae* (68), and *Burkholderiaceae* (69, 70). In contrast, the turquoise, blue, and yellow mOTU modules include halophilic and halotolerant *Rhodobacteraceae* (71) and *Flavobacteriaceae* (72, 73). The presence of *Rhodobacteraceae* suggests a potential for anoxygenic bacterial photosynthesis. Therefore, we connected mOTUs to their corresponding metagenome assembled genomes (MAGs) and quantified how many of them contained *pufM*, an anoxygenic bacterial photosynthesis marker gene (74). Notably, the blue module contains mOTUs with the highest proportion of *pufM*-associated MAGs (12.9%), followed by brown (7.7%) and turquoise (5.71%), while the yellow module-associated MAGs lacked *pufM* entirely. This indicates that taxa in the modules do not have a consensus for energy sources. Instead, modules and their spatial patterns align strongly with the presence of putative osmoregulation genes, occurring in 80%–84% of positively salinity-correlated modules (blue, turquoise, and yellow), and 46%–62.6% of negatively correlated or freshwater-associated modules (gray, green, and brown). This supports our hypothesis that taxa are spatially constrained as an effect of osmoregulation and salinity tolerance.

Freshwater areas have previously been linked to nitrogen cycling. Using WGCNA and indicator analyses, we observed similar salinity-driven localization of nitrogen-associated transcripts to freshwater conditions. These included *UMPS* (*de novo* pyrimidine biosynthesis [75]), *narB* (nitrate assimilation [76]), and *nifD* (nitrogen fixation [77]). The preferential transcription of nitrate assimilation genes in limnetic conditions likely reflects high nitrate concentrations in the Elbe estuary’s freshwater zones (10, 78), driven by remineralization and nitrification near Hamburg Harbor and upstream phytoplankton activity (32). These findings suggest that salinity shifts, such as marine intrusions increased by high dredging activities (79, 80) and droughts, would significantly disrupt estuarine microbiome structure and function. In particular, they may impact freshwater-specific processes, including heterotrophy-autotrophy dynamics, nitrate assimilation, nitrogen accumulation, and *de novo* pyrimidine biosynthesis, ultimately reducing both heterotrophic activity and nitrogen processing capacity.

Dissolved CO_2_ (dCO_2_) declines sharply from freshwater areas to mesohaline zones (Fig. S1), closely correlating with salinity (Pearson, correlation coefficient = −0.67, *P* < 0.01), suggesting that salinity inhibits net dCO_2_ production or consumption in the Elbe estuary. Elevated upstream heterotrophy, most pronounced at the turbidity maximum zone (TMZ), has also been reported (81, 82). This relationship is seasonal, with stronger summer spatial gradients due to enhanced upstream heterotrophy. These patterns are driven by bacterial carbon utilization, as salinity does not affect phytoplankton abundance or production (Fig. S4H), but influences the distribution of specific taxa (83).

Community composition patterns revealed a strong association between the WGCNA-derived green mOTU module and dCO_2_ (Fig. S2 and S3). This module includes diverse taxa, such as *Microcystis* (freshwater cyanobacteria) and *Cyanobium* (low salinity), both phytoplankton (84), highlighting the salinity-dependent heterotrophy-autotrophy balance that shapes dCO_2_ concentrations. Given the dCO_2_ accumulation in freshwater conditions, we considered whether dark CO_2_ fixation pathways may be promoted. Indeed, freshwater conditions experience seasonally independent increases in ATP-citrate lyases (rTCA) functional potential (Fig. S4; ANOVA, eta2 = 0.25, *P*-value = 0.012). Increases that are potentially linked to *Nitrospirota-*driven denitrification, a dominant process in Hamburg Harbor and surrounding marshes (Fig. S5) (85, 86). The harbor itself may confound dCO_2_ concentrations by proximity, as it is a known heterotrophic hotspot (87). Future experiments integrating salinity tolerance with direct activity assays would provide direct mechanistic validation of these sequenced-based inferences.

**Fig 4:**
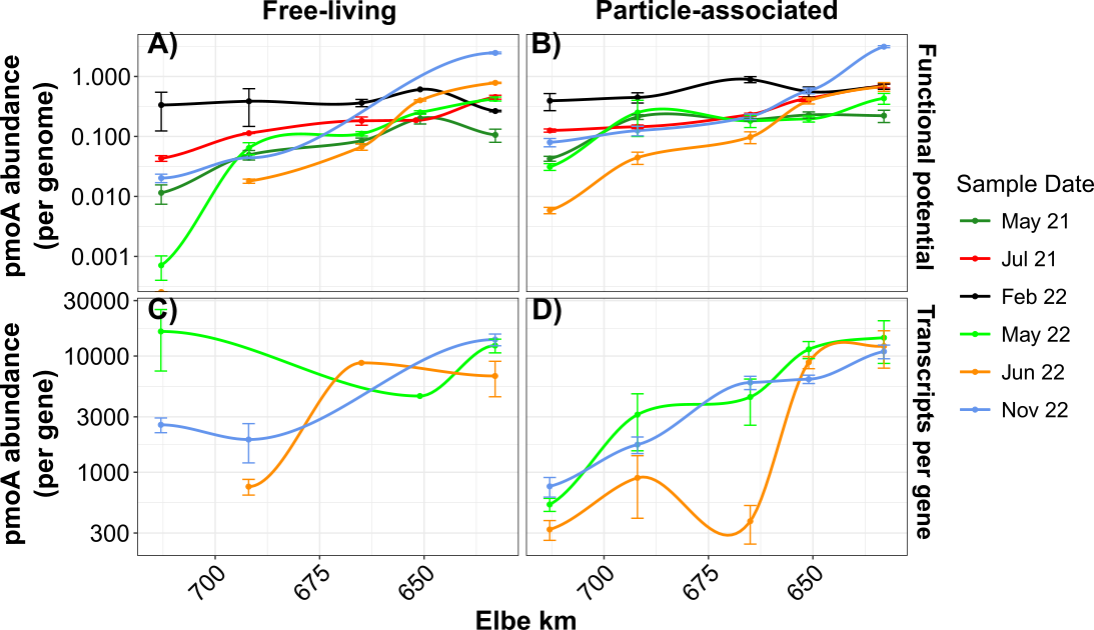
Particulate methane monooxygenase subunit A (*pmoA*) abundance in the Elbe estuary for functional potential per genome and transcription per gene. The mean of two samples, *pmoA* (**A, B**) functional potential and (**C, D**) transcripts per gene, is shown across the Elbe estuary. *pmoA* abundance has been taxonomically corrected against *amoA*. Colors depict sample dates, and standard errors are shown as error bars, and free-living (**A, C**) and particle-associated (**B, D**) abundances have been divided.

Previous studies have shown that estuarine salinization reduces methane emissions (88–90). Our findings support this, showcasing a negative relationship between salinity and dissolved CH_4_ (dCH_4_; Spearman, rho = −0.15, *P* < 0.01; Fig. 2A) on a broad scale. Similarly, particulate methane monooxygenase (*pmoA*) gene abundance per genome (Spearman, rho = −0.49, *P* < 0.01) and transcripts per gene increase as salinity decreases (Spearman, rho = −0.62, *P* < 0.01; Fig. 4). This pattern mirrors methane concentrations and implicates a loss of methanotrophic activity as the cause of methane accumulation. Salinity is known to suppress both methanogenic and methanotrophic taxa (89, 91), explaining the localization of methanotrophs like *Methylococcaceae* (91, 92) and *Methylomonadaceae* (93) to oligohaline conditions (0.5–5 PSU; Fig. S6). Osmolytes may also play a role as a source of variations, previously acting as a carbon source during methanogenesis, but as of now, have been predominantly found in high salinity environments such as salt marshes (94). Osmolytes may also play a role in methylotrophic methanogenesis pathways, as previously shown across salinity gradients with plants (95) and microbes (96), where osmolyte utilization to balance osmotic pressures becomes increasingly important at high salinities.

However, the only dCH_4_-correlated mOTU module (brown) lacked methanogens or methanotrophs, instead consisting largely of *Bacteroidia*, Planctomycetota, and Verrucomicrobiota. The presence of *Saprospirace*ae (97), *Emticicia* (98, 99), and *Novosphingobium* (100) suggests a focus on complex carbon degradation capabilities, rather than direct methane cycling. This, along with the absence of methane-cycling taxa, suggests that dCH_4_ largely originates from the sediment (101), entering the water column via diffusive fluxes, microbubbles, fluvial inflows, and tidal mixing (102–104).

Overall, dCH_4_ reductions in the Elbe estuary are closely linked to salinity, with increased saltwater intrusions and reduced freshwater inflow likely inhibiting sediment methanogenesis and lowering emissions. However, further research is needed to clarify methanogen distributions and their roles in methane production, consumption, and atmospheric exchange; particularly given the multiple CH_4_ sources, including terrestrial runoff, side channels, sediments, and anoxic harbor waters (102–104).

### Exopolymer TEP correlated taxa are salinity specific, unlike CSP

We hypothesized that transparent exopolymer particles (TEP) and Coomassie stainable particles (CSP) significantly influence microbial activity and function due to two main factors: (i) both polymers are produced and modified by phytoplankton and prokaryotes, reflecting bacterial activity and energy investment (25, 105, 106); (ii) TEP, as an extracellular polymeric substance (EPS), offers salinity protection (107), yet its abundance decreases with salinity in Elbe estuary surface waters (16).

Although both exopolymers are abundant, only TEP shows significant associations with microbiome composition (Fig. 2; Mantel, *R* = 0.11, *P* < 0.01), functional potential (Mantel, *R* = 0.16, *P* < 0.01), and transcription (Mantel, *R* = 0.19, *P* = 0.02). The lack of CSP associations may reflect non-uniform microbial interactions with its protein fraction or rapid turnover, as previously proposed (16). TEP’s relationship with the free-living microbiome (Fig. 2) may be linked to the production of EPS by free-living organisms, whereas particle-associated microbes participate in TEP production-utilization cycles that obscure correlations unless taxa are individually assessed (108). Also to consider is that prokaryotes may modify TEP without affecting its abundance (109).

Individual taxonomic analyses identified 148 particle-associated bacterial genera significantly correlated with TEP concentrations (Fig. S7). Despite their low median abundance (<0.8%), these genera peak in freshwater or brackish conditions but are undetectable above 20 PSU (Fig. S7), underscoring the salinity-dependent distribution of TEP-associated microbes in the Elbe estuary. Genomic comparisons reveal that TEP-correlated genomes are significantly enriched in the GH65 and GT51 carbohydrate active enzyme families (glycoside hydrolases and glycosyltransferases). These enzyme families are key factors in EPS turnover and catalyze the hydrolysis, reversible phosphorylation, and/or synthesis of various α-glucosides (typically α-glucobioses or their derivatives [110–112]). Processes are essential for glycosidic bond synthesis and degradation (113), supporting the proposed mechanism for obscured TEP-particle microbiome correlations.

### Particle-associated activity is split into growth vs. methanogenesis focuses

A spatiotemporal analysis approach often overlooks localized drivers such as free-living vs. particle-associated microbiomes or specific particle fractions. Our study addresses these finer-scale dynamics, offering insights into estuarine carbon-processing genes—critical to predicting system responses to ongoing anthropogenic and climate-driven changes.

Taxonomic and functional differences between free-living and particle-associated microbiomes are well established (30, 114, 115). However, most studies focus on specific conditions, such as phytoplankton blooms, single seasons, or individual processes. A broader, multi-seasonal approach is needed to identify consistent functional differences in carbon-associated genes across a highly turbid, mixed estuary.

Our WGCNA identifies one mOTU module (turquoise) that is negatively associated with the free-living fraction. No functional potential modules are associated with the free-living fraction or display similar trends across particle fractions. Indicator species analyses show 49 free-living mOTUs and 257 particle-associated mOTUs (Table S2), yet none appear in the turquoise module. Free-living indicators include *Planktophila* (freshwater bacterioplankton [116, 117]), *Pelagibacter* (marine plankton [118]), and *Rickettsiales* (obligate intracellular-symbionts [119]). Particle-associated mOTUs span a broader taxonomic range, from Verrucomicrobia to Pseudomonadota, prompting a shift in focus toward functional indicators.

While we detect no carbon or nitrogen cycling genes indicative of free-living functional potential, we find 31 genes are indicative of a particle-associated lifestyle (Table S2). These include nitrogen fixation genes (*nifHDK* [120]), plant organic acid growth facilitator *oorAB* (121), methanogenesis-related genes *fdhAB* and *fmdCE* (122–124), and antibiotic degradation and biosynthesis genes (e.g., *bacA, antA*, and *cadA*). Although *nifHDK* does not appear as a transcription indicator, particle microenvironments may mitigate oxygen inhibition of nitrogenases (125, 126), supporting increased net expression. This has important implications for eutrophication, as hypoxia promotes nitrogen retention, which over time can exacerbate estuarine eutrophication (127). The presence of *oorAB* suggests either the presence of plant-associated taxa from terrestrial sources or substantial input of terrestrial plant-derived material (16). In contrast, indicator transcript analyses associate the free-living fraction with antibiotic biosynthesis genes (*andAd* [128, 129]), enzyme 4.1.1.64 (130, 131), and components of the reductive TCA cycle (*fhdF*; Table S2). Contrary to expectations of increased polymer degradation genes in the particle fraction, we observe active carbon turnover in both free-living and particle-associated microbiomes. However, particle-associated communities also engage heavily in nitrogen cycling, while free-living microbes appear under higher competitive stress.

**Fig 5:**
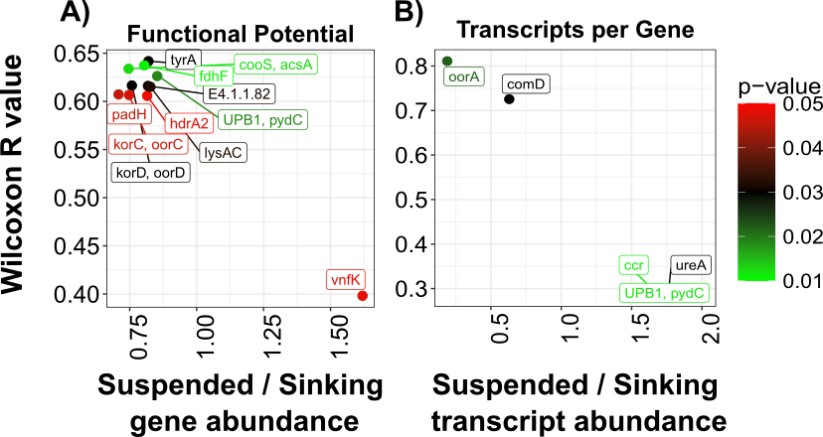
Lifestyle differences between suspended vs. sinking particle-associated functional potential and transcripts per gene. Significantly different functional potential genes (**A**) and transcripts per gene (**B**) are depicted, based on Wilcoxon tests. Color denotes the Mantel *P*-value, with the *y*-axis representing the Wilcoxon *R* value and the *x*-axis representing the ratio between suspended to sinking abundance.

Given the abundance of particle-associated indicator genes, we assessed functional distributions between suspended and sinking particles. Indicator analyses reveal no distinct affiliations, but abundance-based Wilcoxon tests (Fig. 5A) show higher levels of *vnfK* (nitrogen fixation; Wilcoxon, suspended to sinking ratio = 1.6, *R* = 0.40, *P*-value = 0.048 [132]) on suspended particles. Meanwhile, sinking particles exhibit higher expressions of growth-related genes: *korC* (TCA involvement; Wilcoxon, suspended to sinking ratio = 0.75, *R* = 0.61, *P*-value = 0.047 [133]), *tyrA* (amino acid biosynthesis; suspended to sinking ratio = 0.82; Wilcoxon, *R* = 0.64, *P*-value < 0.01 [134]), and *pydC* (pyrimidine degradation; suspended to sinking = 0.85, Wilcoxon, *R* = 0.63, *P*-value = 0.02 [135]). Notably, *hdrA2*, a key methanogenesis intermediate (Wilcoxon, suspended to sinking ratio = 0.82, *R* = 0.61, *P*-value = 0.049 [136]), is enriched in sinking particles, indicating a predisposition to methanogenesis. Suspended particle microbiomes align with our hypotheses, whereas sinking particles lack elevated aromatic compound degradation genes.

Transcript-based indicator analyses find no strong associations for suspended particles. However, Wilcoxon tests (Fig. 5B) link sinking particles with *oorA* (energy involvement; Wilcoxon, suspended to sinking ratio = 0.19, *R* = 0.81, *P*-value = 0.022) and *comD* (coenzyme M biosynthesis, a key methanogenesis cofactor gene; Wilcoxon, suspended to sinking ratio = 0.63, *R* = 0.73, *P*-value < 0.01 [137]). Suspended particles instead show elevated expression of *ccr* (antibiotic synthesis; Wilcoxon, suspended to sinking ratio = 1.7, *R* = 0.29, *P*-value = 0.011 [138]), *ureA* (arginine biosynthesis and xenobiotic degradation; Wilcoxon, suspended to sinking ratio = 1.8, *R* = 0.30, *P*-value = 0.03 [139]), and *pydC* (pyrimidine degradation; Wilcoxon, suspended to sinking ratio = 2.0, *R* = 0.29, *P*-value = 0.011).

In summary, while taxonomic differences between suspended and sinking particle microbiomes are minimal, their functional roles diverge significantly. Suspended particles exhibit active transcription of growth-related pathways, whereas sinking particles are associated with methanogenesis-related pathways.

### Conclusion

The Elbe estuary’s microbiome is shaped by its physicochemical profile, with salinity crucial to understanding carbon gene profiles. Increased marine intrusions are predicted to disrupt freshwater nitrate assimilation, growth processes (e.g., *de novo* pyrimidine biosynthesis), and methane accumulation. Salinity has also been shown to influence TEP and its associated taxa, with detectable associations below 20 PSU. TEP-dependent aggregation diminishes downstream and may lead to reduced carbon sedimentation. Further research is required to determine the extent of marine intrusion effects, including a sediment analysis for microbial community compositions and functions to track methane origins and TEP profiles across depth profiles. Vertical profiles would be especially helpful as dredging plays an important part in aggregation dynamics, and resuspended sediments interact heavily with TEP.

Salinity-independent analysis included the assessment of free-living vs. particle-associated, where particle-associated microbiomes may fix more nitrogen due to increased *nifHDK* gene presence with equal transcripts per gene, and also contain more bacteria of terrestrial origin. Expression patterns further revealed distinct lifestyles between particle fractions, i.e., sinking particle transcripts are linked to methanogenesis, whereas suspended particle-associated microbiomes preferentially transcribe growth-associated genes. Our findings suggest that increased methane concentrations may be related to an abundance of sinking particles through elevated coenzyme M gene expression. This is especially relevant in the highly turbid Elbe estuary, where urban activities (e.g., dredging) greatly affect greenhouse gas emissions, aggregation dynamics, and salinity intrusions. Future studies should examine the underlying mechanisms and controlling variables in greater detail, especially in the context of climate predictions. Our findings highlight potential climate impacts, including methanogen-dependent methane emission decreases and consequent carbon processing gene impairments.

## Data Availability

Raw sequences are available on NCBI under BioProject accession number PRJEB54081 (BioSamples: SAMEA110290250–SAMEA110290357 and SAMEA112714775–SAMEA112714862).

## SUPPLEMENTAL MATERIAL

Supplemental Material msystems.01790-25-s0001.pdf Supplemental figures and captions for supplemental tables.

Figure S7 msystems.01790-25-s0002.pdf A high-definition version of Fig. S7.

Supplemental Methods msystems.01790-25-s0003.docx Detailed report of the sequencing analysis approach.

Table S1 msystems.01790-25-s0004.xls Statistical comparisons between microbiome community composition, functional potential, and gene expression for free-living vs. particle-associated microbiomes.

Table S2 msystems.01790-25-s0005.xlsx Indicator mOTUs, genes, and transcripts across particle fractions.

ASM does not own the copyrights to Supplemental Material that may be linked to, or accessed through, an article. The authors have granted ASM a non-exclusive, world-wide license to publish the Supplemental Material files. Please contact the corresponding author directly for reuse.
