# Supplementary material for: Osmotolerance is a driver of microbial carbon processes in the Elbe estuary
Source: mSystems. 2026 Mar 30;11(4):e01790-25. doi: 10.1128/msystems.01790-25 (PMC13098242; doi:10.1128/msystems.01790-25)
Supplement: Supplemental Material — Supplemental figures and captions for supplemental tables. [file msystems.01790-25-s0001.pdf]

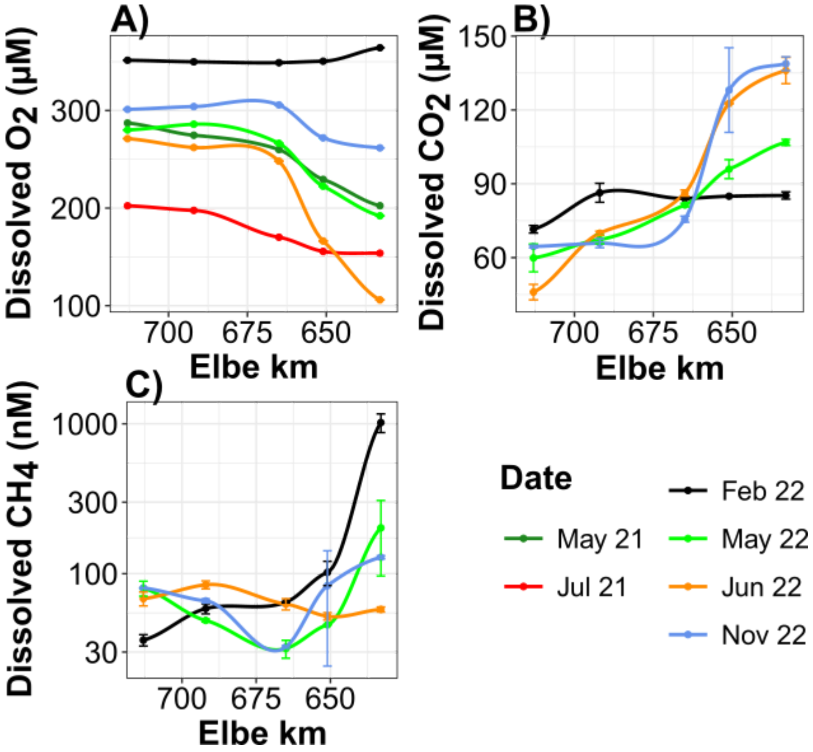

Figure S1. Dissolved gases in the Elbe Estuary. Dissolved **(A)** O_2, **(B)** CO_2, and **(C)** CH_4 are shown across the Elbe Estuary, with colour noting sampling dates. Shaded areas represent the 95% confidence interval.

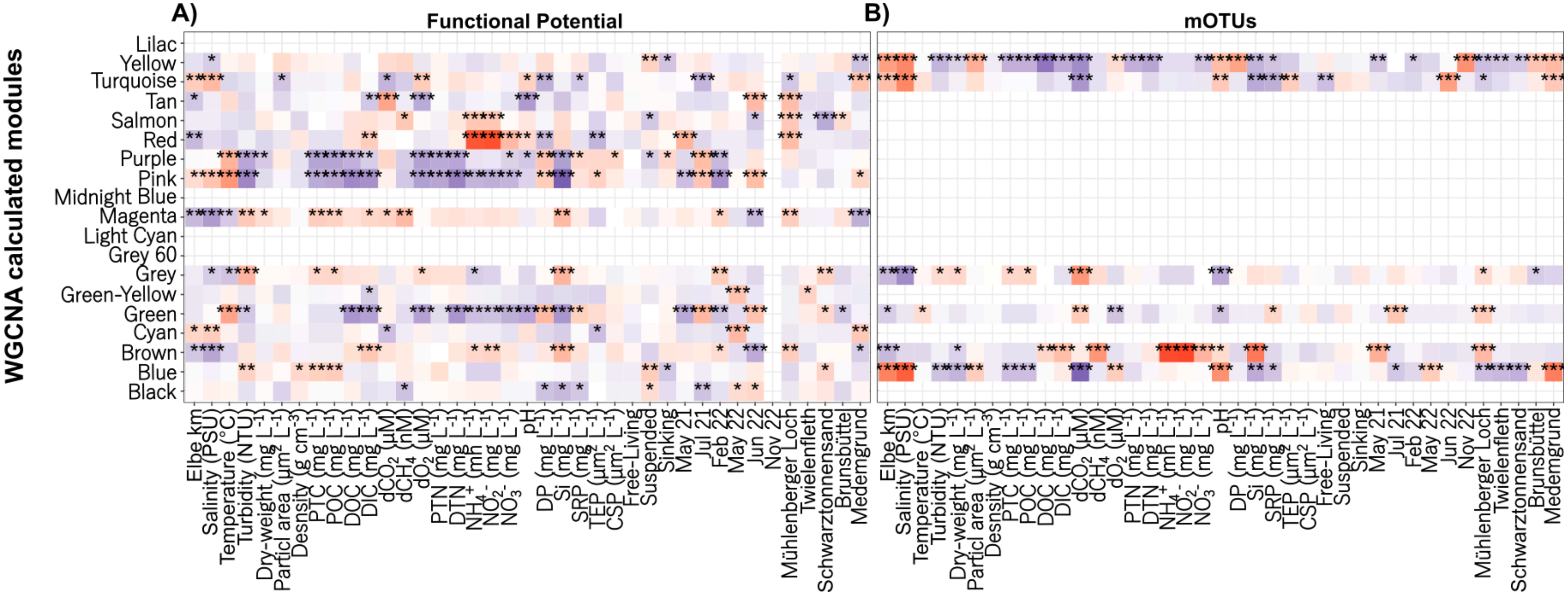

Figure S2. WGCNA network module correlations to physicochemical gradients. mOTU and functional potential underwent Weighted Correlation Network Analysis (WGCNA), clustering genes **(A)** and mOTUs **(B)** into modules based on co-occurrence. Colours represent the direction and strength of the Mantel Pearson correlation (red is positive and blue is negative), while p-value is denoted as stars (" ": non-significant, * < 0.05, ** < 0.01, * < 0.001). WGCNA modules independently calculated for each community aspect.

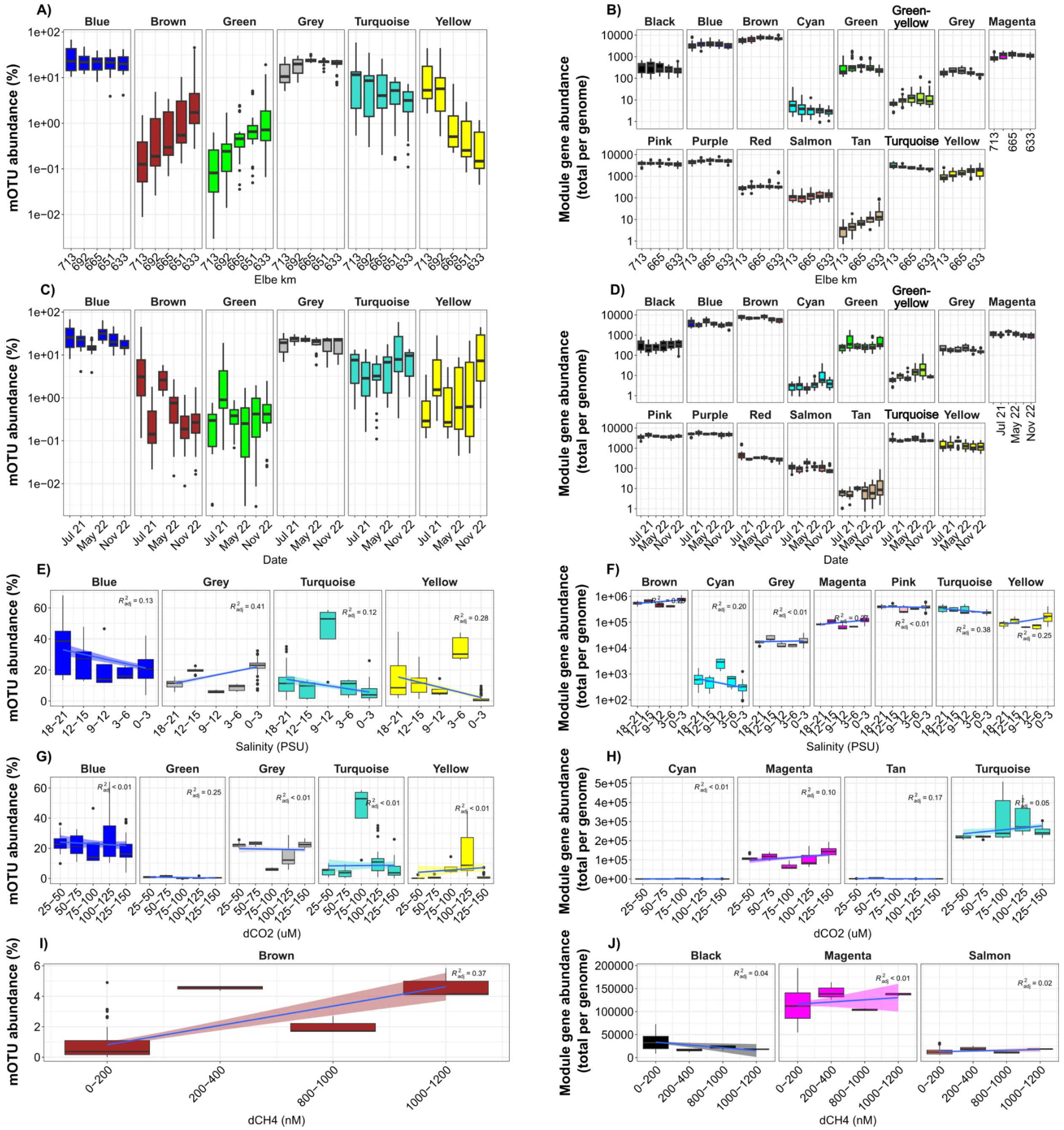

Figure S3. WGCNA module abundance in relation to spatiotemporal and correlated factors. Total module mOTU **(A, C)** and genes per genome **(B, D)** abundance is shown in relation to Elbe km **(A, B)** and sample dates **(C, D)**. Additionally, mOTU **(E, G, I)** and functional potential **(F, H, J)** module abundances are shown against salinity **(E, F)**, dCO_2 **(G, H)**, and dCH_4 **(I, J)** concentrations with a line of best fit adjusted R^2 value.

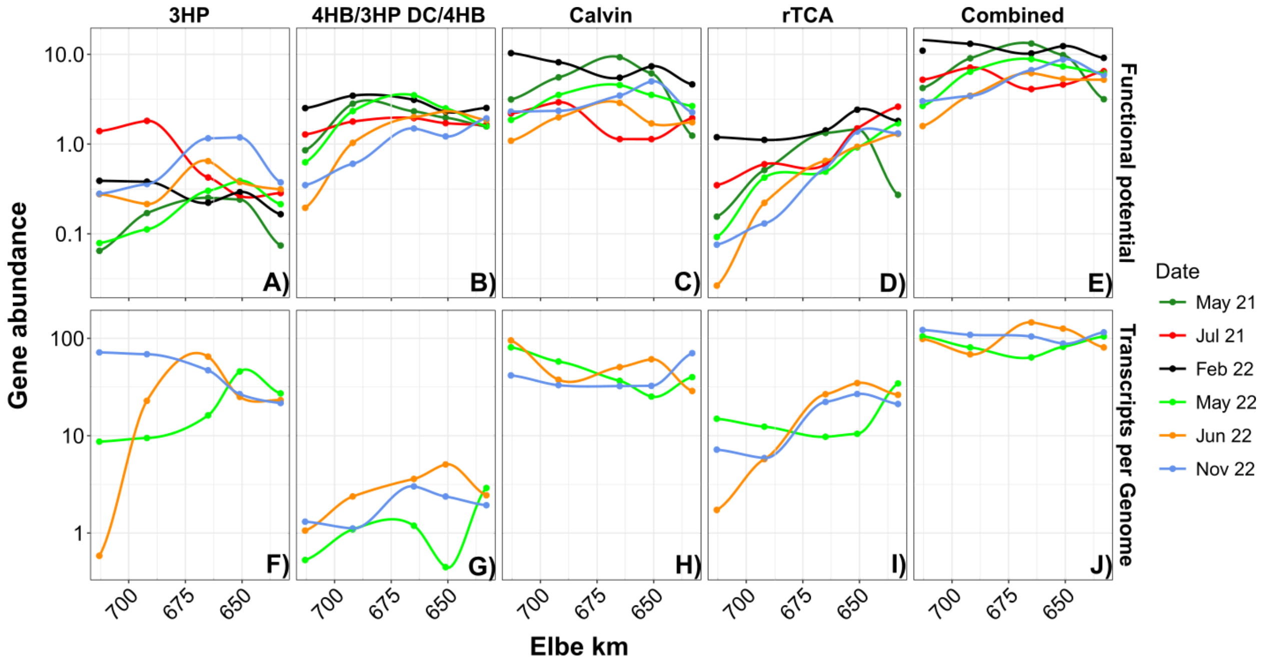

Figure S4. Carbon fixation pathway functional potential and transcription in the Elbe Estuary. The mean of two samples is depicted using different colours for sample dates, for both genes per genome **(A-E)** and transcripts per gene **(F-J)**. Depicted carbon fixation pathways are **(A, F)** the 3-hydroxypropionate (3HP) bi-cycle, **(B, G)** the combined 4-hydroxybutyrate/3-hydroxypropionate (4HB/3HP) cycle and dicarboxylate/4-hydroxybutyrate (DC/4HB) cycle, **(C, H)** the Calvin cycle, **(D, I)** the reverse tricarboxylic acid (rTCA) cycle, and **(E, J)** the sum of the pathways. Since Wood–Ljungdahl pathway—WLP) and reductive glycine pathways operate in both oxidative and reductive directions with identical enzymes (e.g., in methane or acetate oxidation), we are unable to predict the direction and cannot proceed with further analysis.

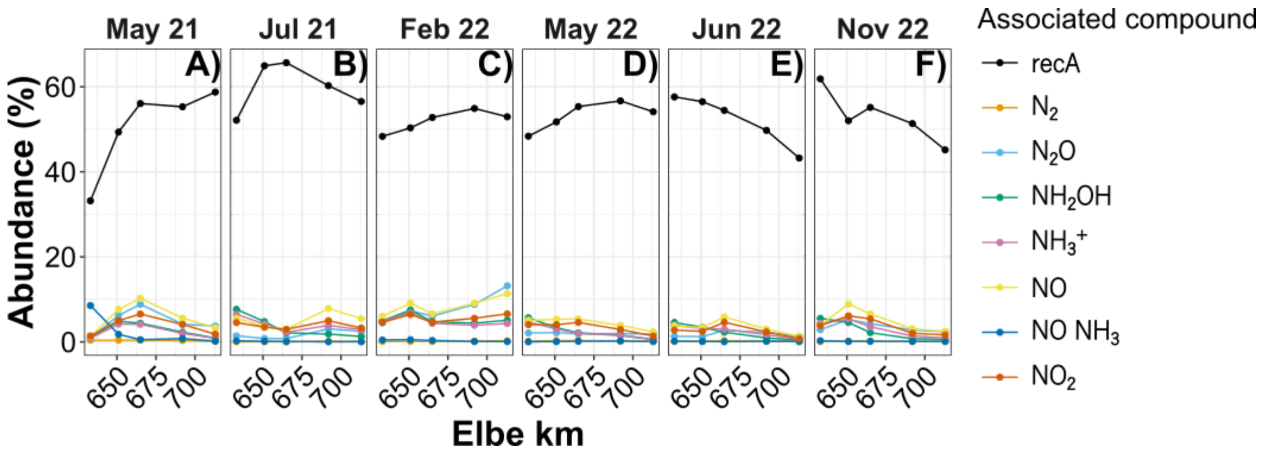

Figure S5. Nitrogen cycling associated gene abundance. The mean of two samples abundance (genome normalised) of genes associated with the processing of the colour dependent compounds.

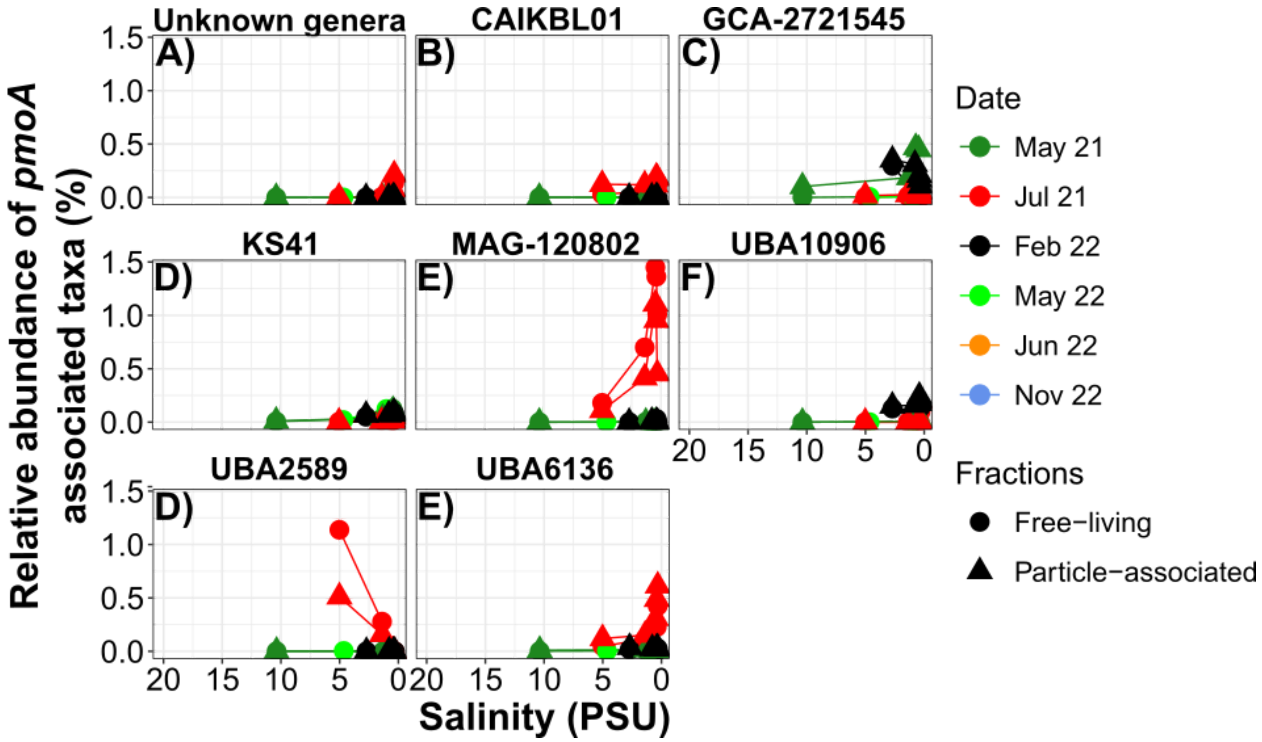

Figure S6. Abundance of *pmoA* correlated taxa. The mean of two samples of *pmoA* correlated taxa are depicted across the Elbe Estuary with colours indicating sample dates. Taxa names are shown above the plot, with taxa abundance shown against salinity as PSU.

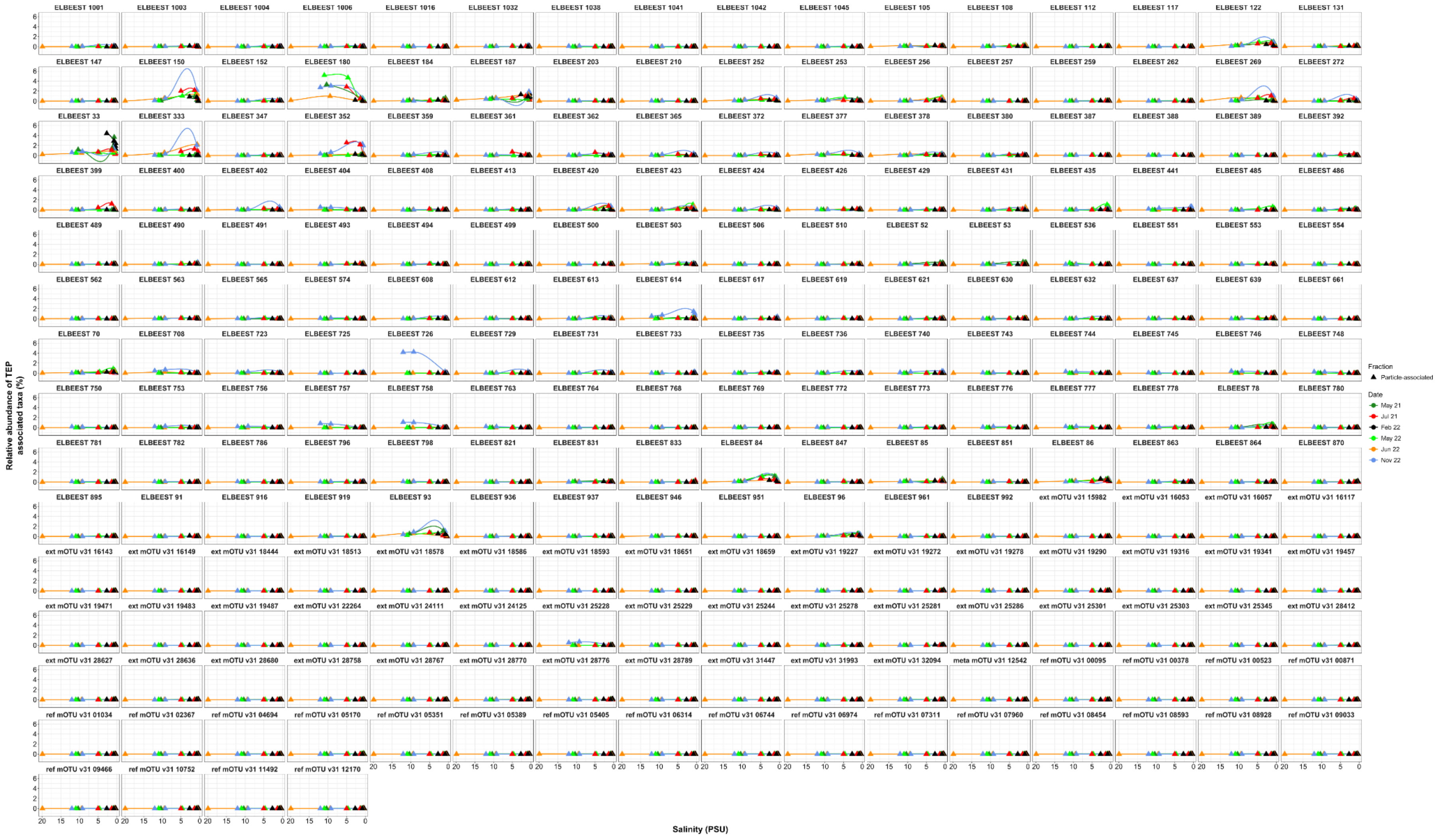

Figure S7. Abundance of TEP correlated taxa. The mean of two samples of TEP correlated taxa are depicted across the Elbe Estuary with colours indicating sample dates. Taxa names are shown above the plot, with taxa abundance shown against salinity as PSU.

Table S1. Statistical comparisons between microbiome community composition, functional potential, and gene expression free-living vs. particle-associated microbiomes. Comparisons are made on a pairwise basis between dissimilarity matrices using the Pearson method. Particle-associated microbiomes include both suspended particle-associated and sinking particle-associated microbiomes, compared against free-living microbiomes.

Table S2. Indicator mOTUs, genes, and transcripts across particle fractions. Differences include free-living vs. particle-associated and suspended vs. sinking particles. Either mOTU taxonomies or gene names and KEGG IDs are included, in addition to test statistics, gene function (if relevant), and relevant citations.
