# Supplementary figures and images for: Osmotolerance is a driver of microbial carbon processes in the Elbe estuary
Source: mSystems. 2026 Mar 30;11(4):e01790-25. doi: 10.1128/msystems.01790-25 (PMC13098242; doi:10.1128/msystems.01790-25)

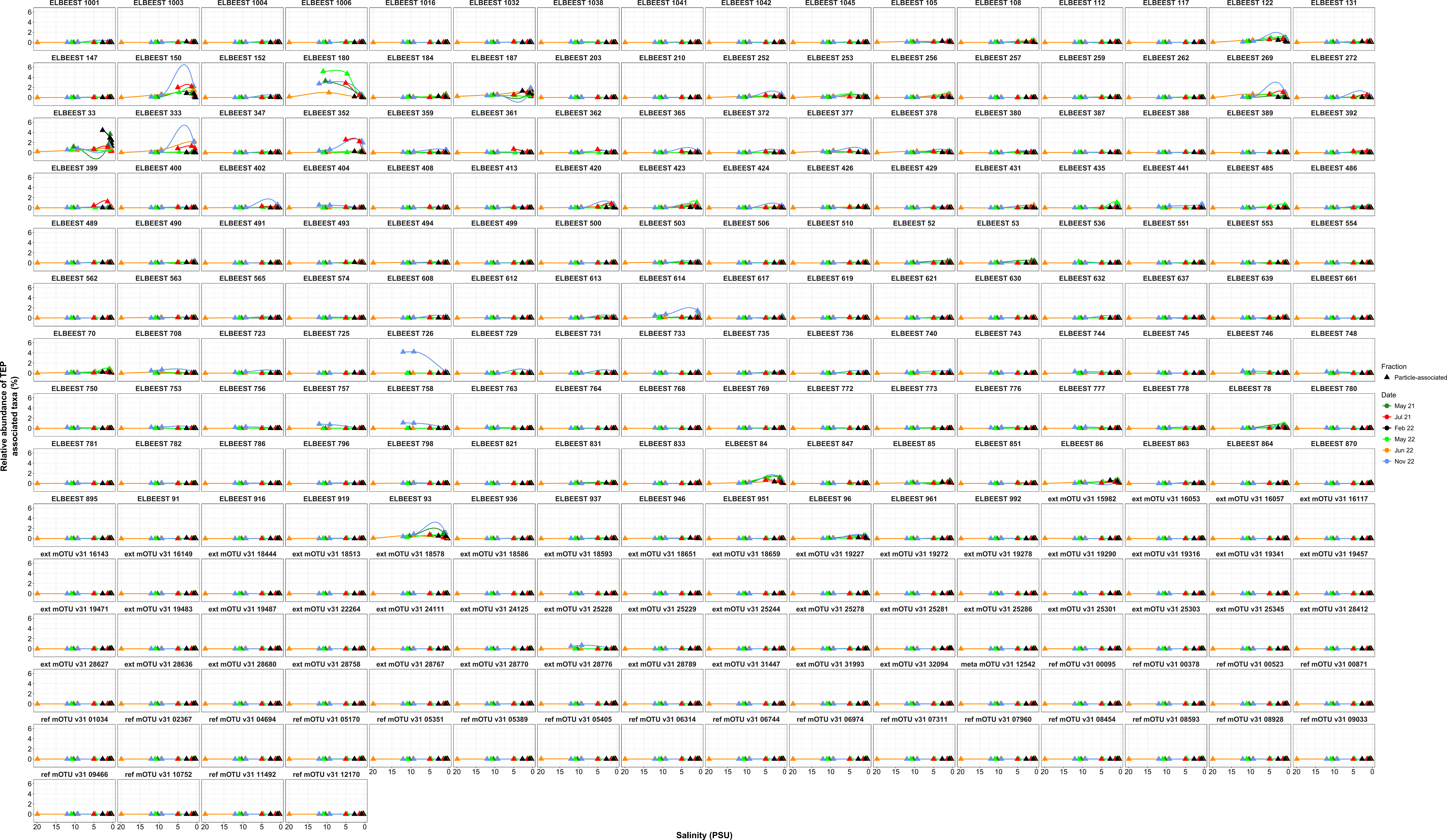

Supplement: Figure S7 — A high-definition version of Fig. S7. [file msystems.01790-25-s0002.pdf]
