# Supplementary material for: Osmotolerance is a driver of microbial carbon processes in the Elbe estuary
Source: mSystems. 2026 Mar 30;11(4):e01790-25. doi: 10.1128/msystems.01790-25 (PMC13098242; doi:10.1128/msystems.01790-25)
Supplement: Supplemental Methods — Detailed report of the sequencing analysis approach. [file msystems.01790-25-s0003.docx]

**Supplementary Methods**

Metagenomic (n=190) and Metatranscriptomic sequence data (n=73) was quality filtered with BBMap (v.38.79; <https://sourceforge.net/projects/bbmap/>), removing sequencing adapters from the reads, reads that mapped to quality control sequences (PhiX genome), and discarding low quality reads using the parameters trimq = 14, maq = 20, maxns = 1, and minlength = 45. Downstream analyses were performed using quality-controlled reads or, if specified, merged quality-controlled reads (bbmerge.sh minoverlap = 16). All metagenomes were assembled individually with metaSPAdes (v.3.15.2; [Nurk et al., 2017)](https://www.zotero.org/google-docs/?pQamgp).

Following assembly, binning for MAG reconstruction individually mapped quality-controlled metagenomic reads from all samples against the scaffolds (≥1 kbp) of at least 80 samples. Reads were mapped with *BWA* (v.0.7.17-r1188; [Li & Durbin, 2009)](https://www.zotero.org/google-docs/?H6up87), allowing the reads to map at secondary sites (with the -a flag). Alignments were filtered to a minimum 45 bases in length, with an identity of ≥97% and covering ≥80% of the read sequence. The resulting BAM files were processed using the *jgi_summarize_bam_contig_depths* script of *MetaBAT2* (v.2.12.1; [Kang et al., 2019)](https://www.zotero.org/google-docs/?HMioZ2) to provide within- and between-sample coverages for each scaffold. The scaffolds were finally binned by running *MetaBAT2* on all samples individually with parameters *--minContig 2000* and *--maxEdges 500* for increased sensitivity.

The quality of each metagenomic bin and external genome was evaluated using both the ‘lineage workflow’ of *CheckM* (v.1.1.3; Parks et al., 2015) and *Anvi’o* (v.7.1; [Eren et al., 2015)](https://www.zotero.org/google-docs/?MlLDCu). Metagenomic bins were retained for downstream analyses according to established best practices [(Ruscheweyh et al., 2022a)](https://www.zotero.org/google-docs/?s6HyQ3), either *CheckM* or *Anvi’o* reporting a completeness/completion (cpl) of ≥50% and a contamination/redundancy (ctn) of ≤10%. Prokaryotic genomes were taxonomically annotated using *GTDB-Tk* (v.2.1.0; [Chaumeil et al., 2020](https://www.zotero.org/google-docs/?QsuCUn)) with the default parameters against the GTDB R214 release [(Parks et al., 2018)](https://www.zotero.org/google-docs/?XqIt3Q). Gene sequences were predicted using *Prodigal* (v2.6.3; [Hyatt et al., 2010)](https://www.zotero.org/google-docs/?lT9z1v) with the parameters *-c -q -m -p single*. rRNA sequences from prokaryotic MAGs were extracted using *Barrnap* (v0.9; [Seemann, 2013/2025)](https://www.zotero.org/google-docs/?7ErpnU) with default parameters.

Viral MAG and gene identifications used *VIBRANT* (v1.3.1; [Kieft et al., 2020)](https://www.zotero.org/google-docs/?RTAFBa) integrated into the *ViWrap* pipeline (v1.3.0; [Zhou et al., 2023)](https://www.zotero.org/google-docs/?49i5zg). A quality check utilised *CheckV* (v1.0.1; [Nayfach et al., 2021)](https://www.zotero.org/google-docs/?f0Tyyw) to only retain high confidence and quality genes. All gene classifications were based on *KEGG* ([Kyoto Encyclopedia of Genes and Genomes;](https://www.zotero.org/google-docs/?uv8uT3) v2019-03-20; [Kanehisa, 2019; Kanehisa et al., 2023; Kanehisa & Goto, 2000)](https://www.zotero.org/google-docs/?xQZY5U), *Pfam* (v32; [Mistry et al., 2021; Paysan-Lafosse et al., 2025)](https://www.zotero.org/google-docs/?ileYOD), and *VOG* (release 94; [Trgovec-Greif et al., 2024)](https://www.zotero.org/google-docs/?yBwMdd) databases, with a minimum scaffold length of 2000 bp.

Gene sequences predicted from prokaryotic MAGs of all samples and gene sequences predicted from Phages were clustered at 95% identity, keeping the longest sequence as representative using *CD-HIT* (v4.8.1; [Fu et al., 2012)](https://www.zotero.org/google-docs/?wO0t6c) with the parameters *-c 0.95 -M 0 -G 0 -aS 0.9 -g 1 -r 1 -d 0.* . Representative gene sequences were aligned against the *KEGG* database (release April.2022) using *DIAMOND* (v2.0.15; [Buchfink et al., 2021)](https://www.zotero.org/google-docs/?xj2auN) and filtered to have a minimum query and subject coverage of 70% and requiring a bitScore of at least 50% of the maximum expected bitScore (reference against itself).

The 190 metagenomes and 73 metatranscriptomes were then mapped to the 5’835’449 million cluster representatives with BWA (v0.7.17-r1188; *-a*; [Li & Durbin, 2009](https://www.zotero.org/google-docs/?6kzmHi)) and the resulting BAM files were filtered to retain only alignments with a percentage identity of ≥95% and ≥45 bases aligned. Length-normalized gene abundance was calculated by first counting inserts from best unique alignments and then, for ambiguously mapped inserts, adding fractional counts to the respective target genes in proportion to their unique insert abundances and dividing the total insert counts by the length of the respective gene. Gene-length normalized read abundances were further converted into per-cell gene copy numbers dividing them by the median abundance of single-copy marker gene copies [(Ruscheweyh et al., 2022b)](https://www.zotero.org/google-docs/?y6Th0m) in each sample.

For taxonomic profiling, the *mOTUs* database (v3.1; [Ruscheweyh et al., 2022)](https://www.zotero.org/google-docs/?rUCyte) was extended with 13765 prokaryotic MAGs forming 1046 new species level clusters. Quality controlled sequencing reads of 259 metagenomes and metatranscriptomes were profiled using the extended mOTUs database with default parameters. MAG dereplication was performed using *dREP* (v3.0.0, -sa 0.99 -nc 0.2; [Olm et al., 2017)](https://www.zotero.org/google-docs/?NoXOvC) yielding 5’017 representative genomes.

**References:**

[Buchfink, B., Reuter, K., & Drost, H.-G. (2021). Sensitive protein alignments at tree-of-life scale using DIAMOND. *Nature Methods*, *18*(4), 366–368. https://doi.org/10.1038/s41592-021-01101-x](https://www.zotero.org/google-docs/?IzcCKH)

[Chaumeil, P.-A., Mussig, A. J., Hugenholtz, P., & Parks, D. H. (2020). GTDB-Tk: A toolkit to classify genomes with the Genome Taxonomy Database. *Bioinformatics*, *36*(6), 1925–1927. https://doi.org/10.1093/BIOINFORMATICS/BTZ848](https://www.zotero.org/google-docs/?IzcCKH)

[Eren, A. M., Esen, Ö. C., Quince, C., Vineis, J. H., Morrison, H. G., Sogin, M. L., & Delmont, T. O. (2015). Anvi’o: An advanced analysis and visualization platform for ‘omics data. *PeerJ*, *3*, e1319. https://doi.org/10.7717/peerj.1319](https://www.zotero.org/google-docs/?IzcCKH)

[Fu, L., Niu, B., Zhu, Z., Wu, S., & Li, W. (2012). CD-HIT: Accelerated for clustering the next-generation sequencing data. *Bioinformatics*, *28*(23), 3150–3152. https://doi.org/10.1093/bioinformatics/bts565](https://www.zotero.org/google-docs/?IzcCKH)

[Hyatt, D., Chen, G.-L., LoCascio, P. F., Land, M. L., Larimer, F. W., & Hauser, L. J. (2010). Prodigal: Prokaryotic gene recognition and translation initiation site identification. *BMC Bioinformatics*, *11*(1), 119. https://doi.org/10.1186/1471-2105-11-119](https://www.zotero.org/google-docs/?IzcCKH)

[Kanehisa, M. (2019). Toward understanding the origin and evolution of cellular organisms. *Protein Science: A Publication of the Protein Society*, *28*(11), 1947–1951. https://doi.org/10.1002/pro.3715](https://www.zotero.org/google-docs/?IzcCKH)

[Kanehisa, M., Furumichi, M., Sato, Y., Kawashima, M., & Ishiguro-Watanabe, M. (2023). KEGG for taxonomy-based analysis of pathways and genomes. *Nucleic Acids Research*, *51*(D1). https://doi.org/10.1093/nar/gkac963](https://www.zotero.org/google-docs/?IzcCKH)

[Kanehisa, M., & Goto, S. (2000). KEGG: Kyoto encyclopedia of genes and genomes. *Nucleic Acids Research*, *28*(1). https://doi.org/10.1093/nar/28.1.27](https://www.zotero.org/google-docs/?IzcCKH)

[Kang, D. D., Li, F., Kirton, E., Thomas, A., Egan, R., An, H., & Wang, Z. (2019). MetaBAT 2: An adaptive binning algorithm for robust and efficient genome reconstruction from metagenome assemblies. *PeerJ*, *7*, e7359. https://doi.org/10.7717/peerj.7359](https://www.zotero.org/google-docs/?IzcCKH)

[Kieft, K., Zhou, Z., & Anantharaman, K. (2020). VIBRANT: Automated recovery, annotation and curation of microbial viruses, and evaluation of viral community function from genomic sequences. *Microbiome*, *8*(1), 90. https://doi.org/10.1186/s40168-020-00867-0](https://www.zotero.org/google-docs/?IzcCKH)

[Li, H., & Durbin, R. (2009). Fast and accurate short read alignment with Burrows–Wheeler transform. *Bioinformatics*, *25*(14), 1754–1760. https://doi.org/10.1093/bioinformatics/btp324](https://www.zotero.org/google-docs/?IzcCKH)

[Mistry, J., Chuguransky, S., Williams, L., Qureshi, M., Salazar, G. A., Sonnhammer, E. L. L., Tosatto, S. C. E., Paladin, L., Raj, S., Richardson, L. J., Finn, R. D., & Bateman, A. (2021). Pfam: The protein families database in 2021. *Nucleic Acids Research*, *49*(D1), D412–D419. https://doi.org/10.1093/nar/gkaa913](https://www.zotero.org/google-docs/?IzcCKH)

[Nayfach, S., Camargo, A. P., Schulz, F., Eloe-Fadrosh, E., Roux, S., & Kyrpides, N. C. (2021). CheckV assesses the quality and completeness of metagenome-assembled viral genomes. *Nature Biotechnology*, *39*(5), 578–585. https://doi.org/10.1038/s41587-020-00774-7](https://www.zotero.org/google-docs/?IzcCKH)

[Nurk, S., Meleshko, D., Korobeynikov, A., & Pevzner, P. A. (2017). metaSPAdes: A new versatile metagenomic assembler. *Genome Research*, *27*(5), 824. https://doi.org/10.1101/GR.213959.116](https://www.zotero.org/google-docs/?IzcCKH)

[Olm, M. R., Brown, C. T., Brooks, B., & Banfield, J. F. (2017). dRep: A tool for fast and accurate genomic comparisons that enables improved genome recovery from metagenomes through de-replication. *The ISME Journal 2017 11:12*, *11*(12), 2864–2868. https://doi.org/10.1038/ismej.2017.126](https://www.zotero.org/google-docs/?IzcCKH)

[Parks, D. H., Chuvochina, M., Waite, D. W., Rinke, C., Skarshewski, A., Chaumeil, P.-A., & Hugenholtz, P. (2018). A standardized bacterial taxonomy based on genome phylogeny substantially revises the tree of life. *Nature Biotechnology*, *36*(10), 996–1004. https://doi.org/10.1038/nbt.4229](https://www.zotero.org/google-docs/?IzcCKH)

[Paysan-Lafosse, T., Andreeva, A., Blum, M., Chuguransky, S. R., Grego, T., Pinto, B. L., Salazar, G. A., Bileschi, M. L., Llinares-López, F., Meng-Papaxanthos, L., Colwell, L. J., Grishin, N. V., Schaeffer, R. D., Clementel, D., Tosatto, S. C. E., Sonnhammer, E., Wood, V., & Bateman, A. (2025). The Pfam protein families database: Embracing AI/ML. *Nucleic Acids Research*, *53*(D1), D523–D534. https://doi.org/10.1093/nar/gkae997](https://www.zotero.org/google-docs/?IzcCKH)

[Ruscheweyh, H.-J., Milanese, A., Paoli, L., Karcher, N., Clayssen, Q., Keller, M. I., Wirbel, J., Bork, P., Mende, D. R., Zeller, G., & Sunagawa, S. (2022a). Cultivation-independent genomes greatly expand taxonomic-profiling capabilities of mOTUs across various environments. *Microbiome*, *10*, 212. https://doi.org/10.1186/s40168-022-01410-z](https://www.zotero.org/google-docs/?IzcCKH)

[Ruscheweyh, H.-J., Milanese, A., Paoli, L., Karcher, N., Clayssen, Q., Keller, M. I., Wirbel, J., Bork, P., Mende, D. R., Zeller, G., & Sunagawa, S. (2022b). Cultivation-independent genomes greatly expand taxonomic-profiling capabilities of mOTUs across various environments. *Microbiome*, *10*(1), 212. https://doi.org/10.1186/s40168-022-01410-z](https://www.zotero.org/google-docs/?IzcCKH)

[Seemann, T. (2013). *barrnap 0.9: Rapid ribosomal RNA prediction* [Computer software]. https://github.com/tseemann/barrnap (Original work published 2013)](https://www.zotero.org/google-docs/?IzcCKH)

[Trgovec-Greif, L., Hellinger, H.-J., Mainguy, J., Pfundner, A., Frishman, D., Kiening, M., Webster, N. S., Laffy, P. W., Feichtinger, M., & Rattei, T. (2024). VOGDB—Database of Virus Orthologous Groups. *Viruses*, *16*(8), 1191. https://doi.org/10.3390/v16081191](https://www.zotero.org/google-docs/?IzcCKH)

[Zhou, Z., Martin, C., Kosmopoulos, J. C., & Anantharaman, K. (2023). ViWrap: A modular pipeline to identify, bin, classify, and predict viral–host relationships for viruses from metagenomes. *iMeta*, *2*(3), e118. https://doi.org/10.1002/imt2.118](https://www.zotero.org/google-docs/?IzcCKH)
